# Intramural Bronchogenic Cysts in the Pediatric Population

**DOI:** 10.7759/cureus.7111

**Published:** 2020-02-27

**Authors:** Sharif Almatrafi

**Affiliations:** 1 Otorhinolaryngology, Head & Neck Surgery, College of Medicine, Prince Sattam Bin Abdulaziz University, Riyadh-Al Kharj, SAU

**Keywords:** intramural, bronchogenic cyst, pediatrics

## Abstract

A bronchogenic cyst is a rare congenital malformation. It occurs due to the development of buds in any part of the tracheobronchial area. It can also lead to fatal complications, especially in the early stages of life. However, data on its diagnosis and treatment are scarce, owing to the rarity of the disease. This review article aimed at evaluating the literature on the manifestations of intramural bronchogenic cysts in the pediatric population. Medical databases were examined thoroughly to explore eligible articles for inclusion. Twenty-three articles appeared in the search result. The produced reports were evaluated against the predecided inclusion criteria. After reviewing the literature, eight articles were eligible for inclusion in this review. The included articles were published between 2000 and 2020. An intramural bronchogenic cyst is a rare condition that should not be neglected in a differential diagnosis. Surgical excision is currently the recommended management strategy. Further extensive studies about the management of the complications of intramural bronchogenic cysts are needed.

## Introduction and background

Bronchogenic cysts have been defined as congenital malformations that are usually coated with epithelium and cilia [1]. The bronchi and their branches start to form in the fifteenth week of fetal development; however, their full development is expected to be completed at eight years of age [2].

Bronchogenic cysts can be detected as early as during gestation, and they are more easily detectable in infants and newborns [3-4]. Yet, mediastinal bronchogenic cysts are usually identified in adulthood [5]. Bronchogenic cysts represent up to 20% of mediastinal lesions and are categorized into intrapulmonary or mediastinal cysts [6].

Approximately three-quarters of patients with bronchogenic cysts have clinical manifestations that could drive the suspicion of the disease [7]. However, almost 90% of patients with mediastinal bronchogenic cysts are asymptomatic, which can result in delayed diagnoses and increased incidence of complications [8].

When present, the most common symptoms associated with bronchogenic cysts resemble symptoms of asthma such as shortness of breath, cough, tachypnea, and wheezing [9]. Sometimes, these symptoms are treated as asthma; however, if the symptoms do not improve, a diagnosis of bronchogenic cysts should be considered and radiological investigations should be ordered [10].

A delayed diagnosis of bronchogenic cysts can lead to the development of complications that can sometimes be life-threatening [11]. Hence, early identification and surgical excision of bronchogenic cysts are mandatory. However, evidence-based data from well-designed articles are scarce due to the disease’s very low prevalence [12]. With these issues in mind, this review aims to examine the literature on the clinical manifestations of intramural bronchogenic cysts in the pediatric patient population.

## Review

An examination of several online databases was conducted, including the PubMed, Google Scholar, and CINAHL indexes. The keywords used included “intramural,” “bronchogenic cyst,” and “pediatrics” to identify relevant publications. The search resulted in 23 articles. Eleven were excluded because they mentioned other types of bronchogenic cysts, and four were excluded based on study design (editorials or short communications). Only eight articles were included; these were published between 2010 and 2020.

Location of bronchogenic cysts

Bronchogenic cysts are usually found in either the pulmonary parenchyma region or the mediastinal region [13]. However, they have also been detected in other anatomical regions, including the abdominal area, diaphragm, neck, retroperitoneum, and skin [14]. The location of the cysts is influenced primarily by the fetal phase when the cyst developed [9,15].

Bronchogenic cysts can develop without any apparent symptoms or complications [3,5,16]. At early developmental stages, bronchogenic cysts are usually located on the tracheobronchial branches [12,17]. Later, however, cysts can be found at peripheral sites and inside the pulmonary parenchyma [18]. When a cyst is symptomatic, patients may feel chest tightness, particularly with effort. This can manifest as an increased left atrial load on electrocardiogram [9,14,19].

Description of bronchogenic cysts

Bronchogenic cysts are covered with cilia on pulmonary secretory epithelium with fibrous tissues, smooth muscle, and mucous glands [8,20]. Cysts contain either air or fluid and can sometimes include both. This is influenced by their connection to the tracheobronchial branches [13,18].

Diagnosis of bronchogenic cysts

A wide range of diagnostic modalities is available for the detection of bronchogenic cysts [2,8]. These techniques are mainly radiological and include magnetic resonance imaging (MRI) and computed tomography (CT) [6,13]. Other invasive diagnostic strategies can be used, such as fine-needle aspiration or excisional tissue examination, which is considered the most accurate diagnostic method [8,20].

On radiological examination of the chest, bronchogenic cysts can appear in three shapes [7,16]. If the bronchi and parenchyma are not interacting, the cysts may be a homogenous lesion with water density or may appear as a bud [9,14].

The formulation of a differential diagnosis is essential in the detection of bronchogenic cysts [4,20]. Alternative diagnoses could include tumors, granulomas, lung sequestration, or vascular defects [3,11].

When a cyst is interacting with the bronchial branches, it appears to contain air or air with fluid. This can lead to a misdiagnosis as lung abscess, carcinoma, or tuberculosis cavities [9,18].

Mediastinal bronchogenic cysts appear as round lesions, with water density [9]. Sometimes, air and fluid or calcification on the peripheral sites can be detected [17]. In that situation, differential diagnosis can include neurological cancers, metastasis, goiter, or lymphoma [18,20].

CT accurately shows the location of the cysts along with their consistency and shape [8,13]. On CT, bronchogenic cysts primarily have a homogeneous texture in addition to water density [19]. However, bronchogenic cysts can have an attenuation of up to 120 Hounsfield units. This is mainly attributed to their protein and calcium content or to infectious complications [9,11,16]. The fluid density can vary as measured on CT, depending on the type of secretion. Additionally, some bronchogenic cysts may contain calcium embedded in the filling fluid and are, therefore, referred to as ''milk of calcium'' [17,20].

On MRI, the fluid inside the cyst can have low or high intensity on T1 images and bright intensity on T2 images [11,19]. In the case of T1-weighted imaging, a white appearance may indicate the presence of hemorrhage, fat, or protein in the field while water has low intensity [18]. On T2 images, water appears to have high density depending on the presence or absence of proteins [16].

Histopathological examination of bronchogenic cysts

When bronchogenic cysts are examined microscopically, they can show some histological features that are found in the bronchi [2,18]. Pseudo-stratification and columnar epithelium with cilia can be observed [16]. Squamous metaplasia can also be present, and cilia can be absent [20].

From a pathological point of view, it could be hard to differentiate an acquired lesion from bronchogenic cysts in the lung [13,19]. This can be attributed to epithelialization of the acquired cysts with other ciliated epithelia [18]. However, the absence of inflammatory signs on the bronchi or history of bronchial inflammation should support the diagnosis of bronchogenic cysts [14,17].

Management of bronchogenic cysts

In the case of asymptomatic patients, the treatment of bronchogenic cysts is still poorly defined, especially in the case of mediastinal cysts [13]. Previous reports demonstrated that surgical removal could be selected in cases where clinical manifestations are present or if there is suspicion for malignancy [11,18].

For other types of bronchogenic cysts, surgical removal should be the preferred treatment [10,18]. This can help reduce the incidence of complications and offers the possibility to confirm the diagnosis through direct tissue examination [20]. Additionally, surgical excision in clinically stable, asymptomatic patients can reduce the risk of perioperative and postoperative complications. Hence, surgical decision-making is recommended as soon as bronchogenic cysts are detected [12].

Surgical excision is usually performed through thoracotomy [11]. Other types of interventions have been described in the medical literature, including sclerosing material injection, transbronchial or percutaneous aspiration, or mediastinoscopy [8,10]. Thoracoscopy has been described as an alternative approach for mediastinal bronchogenic cysts over the past few decades [12]. However, the level of evidence supporting that approach is low, owing to the scarcity of reports examining this technique [13]. The choice of surgical technique should take into consideration the site and size of bronchogenic cysts [8,12]. Cysts that are small and located in the carinal area should be excised as early as possible, especially if they are not connected to any surrounding tissues [4,19]. This can significantly reduce the incidence of complications [5]. Yet, surgical intervention may be associated with complications in cases where cysts are adherent to surrounding tissue, making total excision more challenging [6,19]. In such situations, the remaining part of the cyst can lead to flare-up attacks or other complications in the long term, which favors conservative therapy [14]. The evidence supporting management strategies for such complicated cases suffers from significant deficiencies. This can be attributed to the rarity of the disease, which makes it challenging to carry out well-designed studies to address the gap in the literature [2,4,20].

## Conclusions

Intramural bronchogenic cysts are rare pulmonary lesions that should be approached with caution when identified in the pediatric patient population. Asymptomatic infants with intramural bronchogenic cysts can develop symptoms associated with the progression of the disease and may present with life-threatening complications. Surgical excision, which allows for histopathological diagnosis, is the treatment of choice for intramural bronchogenic cysts. In the presence of symptoms, early surgical intervention can reduce the incidence of surgical complications. There is a pressing need in the literature for well-designed, multicentered, and randomized studies focusing on the sensitivity and specificity of different diagnostic strategies and the optimal management of patients presenting with complications related to intramural bronchogenic cysts.
